# A Rapid and Easy Method of MALDI Biotyper Antibiotic Susceptibility Test Rapid Assay To Provide Early Meropenem Susceptibility Profile in *Enterobacterales*

**DOI:** 10.1128/spectrum.04375-22

**Published:** 2023-01-25

**Authors:** Camila Mörschbächer Wilhelm, Maiara dos Santos Carneiro, Everton Inamine, Afonso Luís Barth

**Affiliations:** a Programa de Pós-Graduação em Ciências Farmacêuticas—Universidade Federal do Rio Grande do Sul, Porto Alegre, Rio Grande do Sul, Brazil; b Laboratório de Pesquisa em Resistência Bacteriana (LABRESIS)—Hospital de Clinicas de Porto Alegre, Porto Alegre, Rio Grande do Sul, Brazil; c Laboratório Carlos Franco Voegeli—Santa Casa de Misericórdia de Porto Alegre, Porto Alegre, Rio Grande do Sul, Brazil; Veterans Affairs Northeast Ohio Healthcare System

**Keywords:** MBT-ASTRA, MALDI-TOF MS, relative growth, antimicrobial susceptibility, MALDI-TOF, mass spectrometry

## Abstract

Antimicrobial susceptibility testing (AST) that can provide faster results is necessary. The MALDI Biotyper antibiotic susceptibility test rapid assay (MBT-ASTRA) can provide early AST results but still needs to be simplified in order to facilitate its execution by microbiology laboratories. The aim of this study was to evaluate an adaptation of MBT-ASTRA. Isolates of *Enterobacterales* were tested for meropenem susceptibility by MBT-ASTRA using a solution prepared from meropenem disks and performing a manual spectrum analysis. The relative growth (RG) was calculated for each isolate, and a cutoff value was established to determine the susceptibility profile of the isolates. Results of the adapted method were compared with the standard susceptibility method (broth microdilution). An adapted method of MBT-ASTRA was developed. The RG cutoff values for meropenem were ≤0.1510 for susceptibility and >0.6272 for resistance, presenting 95.65% categorical agreement, with 2.9% (2/69) minor discrepancy and 3.23% (1/31) very major discrepancy. MBT-ASTRA can be used to provide rapid AST results with a simpler and more accessible protocol, especially regarding spectrum analysis.

**IMPORTANCE** The simplification of the MBT-ASTRA technique, especially in spectrum analysis, can considerably allow more laboratories to rapidly determine antimicrobial susceptibility profiles.

## INTRODUCTION

Carbapenem-resistant *Enterobacterales* (CRE) are probably the main threat for public health. The major CRE resistance mechanism, carbapenemase production, can be easily spread through mobile genetic elements and is related to increased mortality rates (1–4). Moreover, inappropriate antibiotic treatment of CRE is around 25%, which can be associated with high mortality rates (4–7). These poor outcomes of patients infected by CRE are also due to a delay in reporting antimicrobial susceptibility testing (AST) results. Therefore, methodologies that can provide early AST are necessary to improve patient treatment.

Assays to provide early AST results have been developed, such as the EUCAST rapid antimicrobial susceptibility testing (RAST) for positive blood cultures, which is based on the standard disk diffusion methodology with some modifications, such as shortened incubation times (4, 6, and 8 h) (8). Additionally, a shortened disk diffusion method from bacterial colonies has been evaluated, but this methodology still requires at least 6 h of incubation (9, 10). Methodologies that could provide AST results in an even faster turnaround time have been proposed with the use of matrix-assisted laser desorption ionization–time of flight (MALDI-TOF) mass spectrometry (MS) (11–13).

MALDI-TOF MS is primarily used in microbiology laboratories with the purpose of microorganism identification. This methodology is based on the evaluation of a mass spectrum of bacterial proteins, which is compared to a database and provides the species identification according to spectral similarity (14, 15). Since a minimum of bacterial biomass is required for the spectrum acquisition by MALDI-TOF, a very small amount of bacteria may not be sufficient to provide satisfactory results. The MALDI Biotyper antibiotic susceptibility test rapid assay (MBT-ASTRA) (11) was based on the principle that the bacterium in the presence of the antibiotic would not generate enough biomass.

The MBT-ASTRA evaluates the area under the curve (AUC) of the peaks of the spectrum of the bacterium incubated with antibiotic (AUC_BAC+ATB_) in comparison to the area under the curve of the peaks of the spectrum of the bacterium incubated without antibiotic (AUC_BAC_). The ratio (AUC_BAC+ATB_)/(AUC_BAC_) provides a value termed relative growth (RG). An RG value close to 0 (zero) indicates that there was no significant bacterial growth in the tube containing antibiotic, probably due to its action against the bacterium, indicating susceptibility. On the other hand, an RG close to 1 indicates that the bacterium grew in the tube with antibiotic in a similar way as in the tube without antibiotic, indicating resistance. The main advantage of the MBT-ASTRA is the fast analysis of the spectra obtained, providing a rapid AST result in approximately 2 to 3 h (11). Although spectral analysis is performed very quickly, the software commonly used requires programming expertise (16), which is not usually available to professionals in microbiology laboratories in most countries. The company Bruker Daltonics has developed a prototype software program, but it is not currently available. Thus, the development of an adaptation of this assay is of great interest, in particular for hospital laboratories that are aiming to provide faster AST results.

The objective of this study was to evaluate an adaptation of the MBT-ASTRA, exploring ways to facilitate the execution of the method, especially regarding spectral analysis, in order to provide an easy and faster method.

## RESULTS

The control strain Escherichia coli ATCC 25922, which was tested in duplicates by broth microdilution with four solutions prepared with meropenem disk elution (2 after 10 to 15 min and 2 after 2 h of elution), presented the same MIC, 0.03 mg/L, for all preparations.

**FIG 1:**
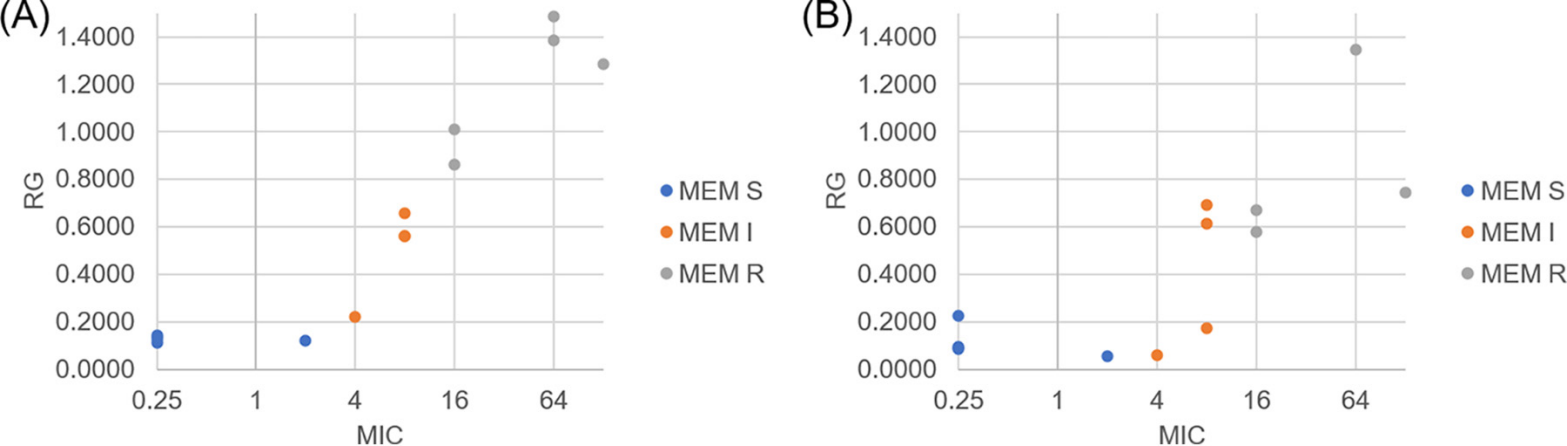
RG values plotted against MICs. Isolates were tested with 8 mg/L (A) and 16 mg/L (B) of meropenem (MEM) solution.

Initially, 13 isolates were tested for 8- and 16-mg/L meropenem solutions, and the RG value was calculated with raw spectra and intensity parameter. The concentration that better separated, by visual analysis, the isolates as susceptible (S), intermediate (I), and resistant (R) was 8 mg/L (Fig. 1). Therefore, the meropenem solution of 8 mg/L was established as the standard for all the experiments with a total of 69 isolates of *Enterobacterales* with different MICs of meropenem and a variety of carbapenemases (Table 1).

**TABLE 1:** Characteristics of the tested isolates and MBT-ASTRA results^*b*^

Species	Meropenem MIC (mg/L)	EUCAST interpretation	qPCR-HRM	No. of isolates (*n*)	No. with MBT-ASTRA result		
					S	I	R
Enterobacter bugandensis	≤0.25	S	NT	2	2	0	0
Enterobacter cloacae	≤0.25	S	NT	1	1	0	0
	1	S	NT	1	1	0	0
Enterobacter hormaechei	4	I	*bla*_KPC_	1	0	1	0
Enterobacter spp.	2	S	*bla*_OXA-48-like_	1	1	0	0
	>64	R	*bla*_KPC_	1	0	0	1
Escherichia coli	≤0.25	S	NT	4	4	0	0
	1	S	*bla*_NDM_	1	1	0	0
	4	I	*bla*_NDM_	1	0	1	0
	32	R	*bla*_NDM_	1	1^*a*^	0	0
	>64	R	*bla*_KPC_	1	0	0	1
Klebsiella aerogenes	≤0.25	S	NT	2	2	0	0
Klebsiella pneumoniae	≤0.25	S	NT	6	6	0	0
	≤0.25	S	Negative	1	1	0	0
	2	S	Negative	2	2	0	0
	4	I	Negative	2	1^*a*^	1	0
	8	I	Negative	2	0	2	0
	8	I	*bla*_NDM_	3	0	3	0
	16	R	Negative	1	0	0	1
	16	R	*bla*_NDM_	1	0	0	1
	32	R	*bla*_KPC_	4	0	0	4
	32	R	*bla*_NDM_	1	0	0	1
	64	R	*bla*_KPC_	6	0	0	6
	64	R	*bla*_NDM_	1	0	0	1
	>64	R	*bla*_KPC_	7	0	0	7
Morganella morganii	≤0.25	S	NT	2	2	0	0
	2	S	*bla*_NDM_	1	1	0	0
Proteus mirabilis	≤0.25	S	NT	1	1	0	0
Serratia marcescens	≤0.25	S	NT	4	3	1^*a*^	0
	32	R	*bla*_KPC_	2	0	0	2
	64	R	*bla*_KPC_	1	0	0	1
	>64	R	*bla*_KPC_	4	0	0	4

Total				69			

a Divergent results in relation to the reference method.

b qPCR-HRM, high-resolution melting real-time PCR; S, susceptible, standard dosing regimen; I, susceptible, increased exposure; R, resistant; NT, not tested.

**FIG 2:**
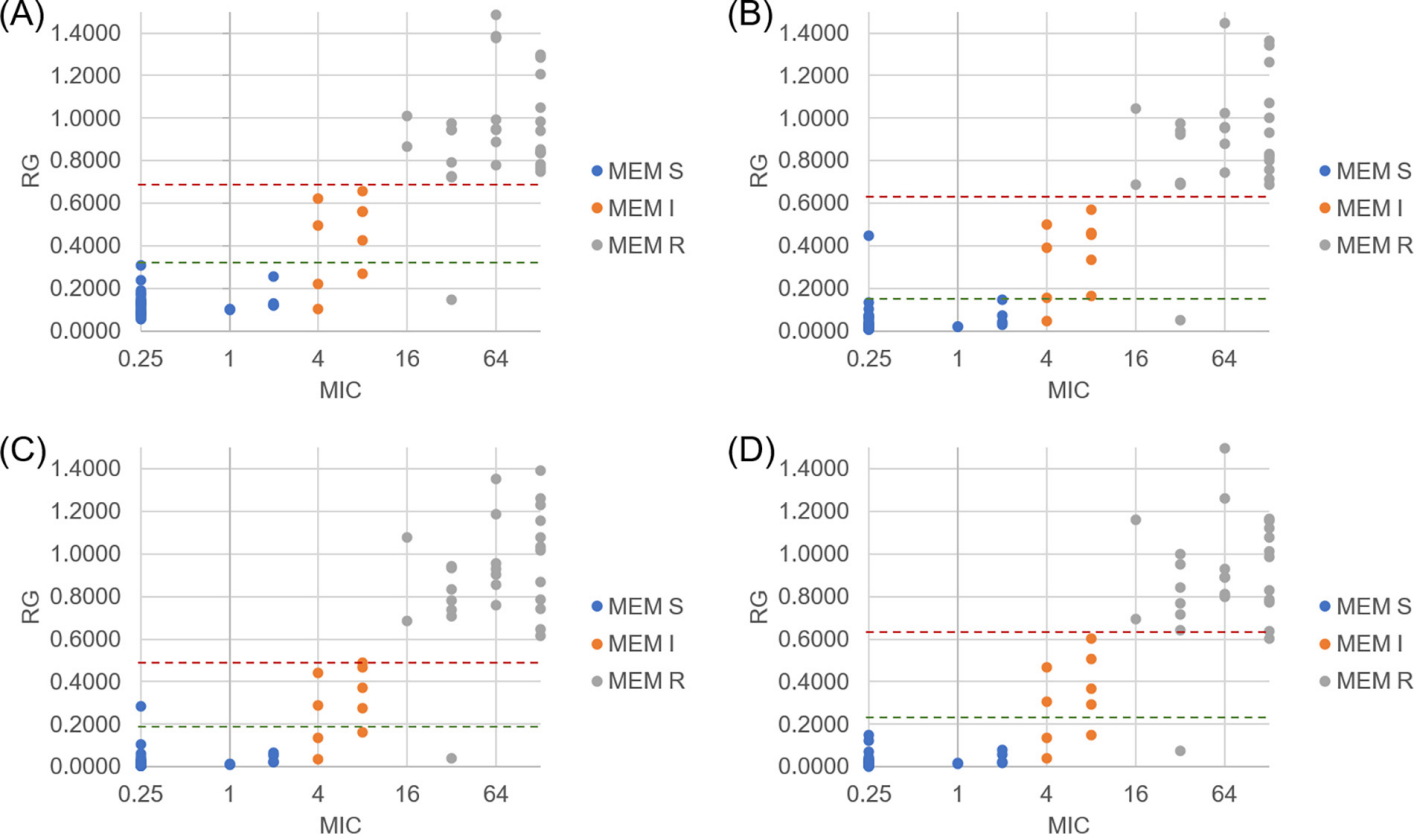
Relative growth (RG) values are plotted against MICs. Panels A and B demonstrate RG values calculated with intensity, while panels C and D represent RG values calculated with AUC. Panels A and C were obtained with raw spectra, and panels B and D were obtained with processed spectra. Dashed green lines indicate RG cutoff value for “susceptible, standard dosing regimen” (S), and dashed red lines indicate RG cutoff value for “resistant” (R); the isolates in between are considered “susceptible, increased exposure” (I).

The experiments performed with 8 mg/L meropenem were used to determine whether intensity and AUC can provide the same results for RG value, as well as to assess whether it is possible to use the raw data or whether it is necessary to process spectra. Visual inspection of the graphs of RG versus MIC values indicated that there was no significant difference using either the intensity of peaks or the AUC to calculate the RG. Moreover, the RG values were also very similar using either raw data or processed spectral data (Fig. 2).

In order to confirm the results obtained by visual inspection, we have used the receiver operating characteristic (ROC) curve to establish the best cutoff value to classify isolates into S, I, and R categories for each combination of parameters used for RG calculation (intensity or AUC) and spectral processing (raw or baseline subtracted and smoothed). Considering broth microdilution as the reference method, the cutoff values that presented better sensitivity and specificity, as well as the highest area under the ROC curve, were the ones determined for the combination of intensity and processed spectra (Table 2).

**TABLE 2:** Relative growth (RG) cutoff values for each combination of parameter and processing spectrum analyzed^*a*^

Parameter	Spectrum processing	Cutoff value for:		Sensitivity (%)	Specificity (%)	ROC curve^*b*^	CA (%)
		S	R				
Intensity	Raw	≤0.2617		96.6	92.5	0.976	89.86
			>0.6878	100	96.8	0.987	
	Processed	≤0.1510		96.6	95.0	0.981	95.65
			>0.6272	100	96.8	0.986	

AUC	Raw	≤0.2876		96.6	87.5	0.954	89.86
			>0.4781	94.7	96.8	0.963	
	Processed	≤0.2209		96.6	90.0	0.958	92.75
			>0.6215	97.4	93.5	0.965	

a AUC, area under the curve; S, susceptible, standard dosing regimen; R, resistant; CA, categorical agreement.

b Area under the receiver operating characteristic (ROC) curve.

According to the cutoff values for S and R determined by the combination of intensity and processed spectra (Table 2), the categorical agreement was 95.65% (66/69), with 2.90% (2/69) minor discrepancy, no major discrepancy, and 3.23% (1/31) very major discrepancy (Table 1). Therefore, one isolate was classified as S by the MBT-ASTRA and was classified as I according to the reference method; it was a Klebsiella pneumoniae isolate that presented an RG value of 0.0470 and a meropenem MIC of 4 mg/L. One isolate, identified as Serratia marcescens, was determined as I by MBT-ASTRA, while it was S by the reference method, with an RG value of 0.4492 and a MIC of ≤0.25 mg/L. Finally, the one isolate, an E. coli isolate, that was categorized as S but was actually R by the reference method, presented an RG value of 0.0488 and a MIC of 32 mg/L. These three isolates were repeated for MBT-ASTRA, presenting RG values of 0.0703, 0.1593, and 0.0498, respectively.

Considering intensity and processed spectra for the following interpretations of our results and disregarding the isolates misclassified by MBT-ASTRA, S isolates presented RG values from 0.0066 to 0.1451, I isolates presented RG values from 0.1571 to 0.5696, and R isolates had RG values from 0.6849 to 1.8374, except for one isolate that presented a very high RG value (RG = 5.0306). This isolate was retested and demonstrated an RG value of 1.4474. Additionally, there was a strong correlation between RG and MIC, as determined by Spearman’s test (*P* < 0.001; ρ = 0.858). RG values recalculated with 4 peaks presented, according to ROC curve analysis, cutoff values of S of ≤0.1462 and R of >0.6089, with the same sensitivity and specificity as results calculated with 6 peaks.

## DISCUSSION

It has already been demonstrated that MALDI-TOF MS technology can effectively improve microbiology diagnosis by reducing the time for microorganism identification (15). Methods using MALDI-TOF MS to reduce the time for AST results were also developed, but it is still necessary to make them accessible for routine microbiology laboratories. MBT-ASTRA is one of the most promising methods that can rapidly provide an AST result. There are several steps in MBT-ASTRA, which can be detailed by the following steps: antibiotic solution preparation, inoculum preparation, incubation, protein extraction, spectrum acquisition, spectrum analysis (spectrum processing—baseline subtraction, normalization to the maximum peak, relative intensity between 0 and 1 subdivided into 0 and 100 equally spaced thresholds, peak picking, counting of the peaks above each threshold, plotting of the resulting number of peaks against the relative intensity threshold, and determination of the AUC for each spectrum), determination of RG value for each isolate, and interpretation of RG values (11). The aim of this study was to simplify these steps, in particular, spectrum analysis.

An important step is the preparation of meropenem solution. We prepared the solution by eluting meropenem from a disk (Oxoid Thermo Scientific) in the same way that some methods have already successfully proposed the use of antibiotic elution, such as colistin broth disk elution (17), carbapenem inactivation method (CIM) (18), and its modified method (mCIM) (19), which used 30 min, 2 h, and 4 h of elution time, respectively. We have used 10 to 15 min to elute the antibiotic, and we found that the meropenem solutions prepared with this reduced time presented the same results in comparison to 2 h of elution for the quality control E. coli ATCC 25922. It is noteworthy that all solutions provided a meropenem MIC of 0.03 mg/L, which is the target for internal quality control as recommended by EUCAST (target, 0.016 to 0.03 mg/L; range, 0.008 to 0.06 mg/L) (20). This indicates that the quantity of meropenem eluted in 10 to 15 min was the same as that in 2 h and that possibly all meropenem content was eluted, validating the use of the meropenem disk as a source of meropenem solution.

Previous studies that assessed MBT-ASTRA from bacterial colonies demonstrated that this assay can be applied to determine antimicrobial susceptibility. Important results were found for the combinations meropenem-Klebsiella pneumoniae, meropenem-Klebsiella oxytoca, cefotaxime-Escherichia coli, tobramycin-K. pneumoniae, meropenem-Pseudomonas aeruginosa, tobramycin-P. aeruginosa, and tobramycin-Acinetobacter baumannii (11, 12). Although there were no nonfermenters among our isolates, we were able to test other species, such as Enterobacter spp., E. coli, Morganella morganii, Proteus mirabilis, and Serratia marcescens. We chose meropenem because it is one of the most used carbapenems and a reduced susceptibility to this antibiotic can suggest the production of carbapenemase. In fact, according to EUCAST, meropenem presents the best specificity and sensitivity to be used as a screening method to detect carbapenemases (21).

Similarly to the work of Lange et al. (11), we found the use of a meropenem concentration of 8 mg/L in the MBT-ASTRA technique provided results that would better differentiate the isolates as S, I, and R. It is important to mention that the concentration used in MBT-ASTRA is different from the breakpoint determined by EUCAST. We also found important the use of a higher concentration of RNase B (20 g/L), the internal standard, to better visualize the peaks in the spectra which allowed their manual selection for analysis in flexAnalysis software. The addition of an internal standard allows a relative quantification to be performed as the peak of interest is first compared to the internal standard peak intraspectrum, allowing the spectra of different samples to be compared. It is important to note that the internal standard must be added to all samples and its concentration must be constant (22, 23). Considering the fact that MALDI-TOF MS can present small variabilities, which are intrinsic to the technique (22), we suggest no fewer than 4 spots for testing each sample, as also suggested by other authors (11). Furthermore, by using the median of the sums of the peak intensities, the highest and the lowest values will be discarded for the RG calculation, which also contributes to minimizing inherent MALDI-TOF MS variabilities.

An important advantage of the MBT-ASTRA technique is the use of the same parameters applied for microbial identification, usually named the MBT_FC.par method, for spectrum acquisition. In our experience, each MALDI-TOF mass spectrometer needs adjustments in order to obtain the same result. Therefore, by applying the parameters for microbial identification, possibly no further configuration is necessary if these parameters are properly functioning and are quality control checked according to the manufacturer.

Spectrum analysis of MBT-ASTRA was performed in other studies using the R language with the MALDIquant package (11, 12, 16, 24) or by the MBT-ASTRA prototype software (Bruker Daltonics), written in R language (25, 26). As the use of R language requires programming expertise and the MBT-ASTRA prototype software is not freely or commercially available, we performed, when applicable, spectrum processing, i.e., baseline subtraction and smoothing, using flexAnalysis software. Spectra are processed in order to minimize MALDI-TOF MS variabilities, such as background or chemical noise, and the baseline subtraction reduces the noise that could affect peak intensity and AUC of the analytes. However, when the intensity or AUC is very low, the analyte could also be subtracted from intensity value and AUC (22, 27, 28). Although previous studies of MBT-ASTRA (11, 12, 24, 26, 29) evaluated only processed spectra, we found it important to verify if baseline subtraction and smoothing could interfere with results, and we confirmed that spectrum processing actually provided better results but, however, only when intensity was considered for RG calculation. Accordingly, we believe that it is better to use processed spectra in order to minimize variabilities of the method. Furthermore, previous studies (11, 12, 24, 26, 29) of MBT-ASTRA used AUC for RG calculation. We found that when the peak intensity was applied in the algorithms, consistent and reliable results were obtained. Additionally, an advantage of using peak intensity instead of AUC is that the intensity can always be measured in flexAnalysis software, in contrast to the AUC, which may not be evaluable if the AUC is too small.

Lange et al. (11) determined by visual inspection the RG cutoff of 0.4 for meropenem to distinguish susceptible and resistant isolates (susceptible RG of <0.4 and resistant RG of ≥0.4). Other studies (12, 24–26, 29), testing other antibiotics, have also applied the same value and concluded that this cutoff was capable of distinguishing susceptible from intermediate/resistant isolates. Currently, EUCAST has redefined the “old” intermediate category as “susceptible, increased exposure,” and consequently, the I isolates can no longer be grouped with the R category. In order to categorize isolates as S, I, and R, we applied ROC curve analysis, which provided satisfactory cutoff values, with good sensitivity and specificity. We believe that each laboratory, when validating this in-house methodology, should determine its own cutoff points, correlating with the MIC as performed in their routine according to the method, such as broth microdilution or gradient diffusion.

Interestingly, we found RG values above 1, meaning that the bacterium grew more in the presence of the antibiotic than in its absence. This situation would not normally be expected. However, Roth et al. demonstrated that, due to uncertain modifications in *bla*_KPC_ expression, an increase in MIC for some antibiotics can occur in some species in the presence of subinhibitory concentrations (30). However, the exact mechanism that induces this situation is still unclear.

Although previous studies (11, 12) performing MBT-ASTRA did not find a direct correlation between MIC and RG values, Jung et al. (24) described a possible correlation for ciprofloxacin. In order to asses a possible correlation, we applied the nonparametric Spearman test, which indicated a strong correlation (*P* < 0.001; ρ = 0.858) between meropenem MIC and RG values.

Detection of carbapenemase genes by quantitative PCR (qPCR) (considered a standard method) could possibly be faster than or require the same time to be executed as the MBT-ASTRA. Even though qPCR can be more specific, it does not detect meropenem resistance due to resistance mechanisms other than specific carbapenemases. Moreover, qPCR results cannot always be related to the MIC, as some meropenem-susceptible isolates are carbapenemase producers.

Although more studies with other combinations of species and antibiotics are needed, we demonstrated that MBT-ASTRA can be used to provide early AST results. The adapted assay facilitates its execution by using antibiotic solution prepared with disk elution, using a common incubator without agitation, and calculating RG values in a simpler way. The manual spectrum analysis can increase time to obtain the AST result, but the result can still be reported in around 3 to 4 h. Furthermore, this method can be optimized for other antibiotics, considering variations in the volume of bacterial inoculum, antimicrobial concentration, and time of incubation. After in-house validation of this method, a control strain, such as E. coli ATCC 25922, can be applied as quality control, which should be categorized as S.

We were able to demonstrate that MBT-ASTRA can be adapted, with simplification of some steps, facilitating its application by routine microbiology laboratories. This assay can lead to a decrease in the time for reporting an AST result, which can be very significant for adjusting therapy and fighting infections.

## MATERIALS AND METHODS

### Bacterial strains.

A total of 69 clinical isolates of *Enterobacterales*, obtained from several anatomical sites from 2019 to 2022, stored at the bacterial bank of the Laboratório de Pesquisa em Resistência Bacteriana (LABRESIS), were selected by convenience according to various meropenem MICs that had previously been determined by the gradient diffusion method in the routine microbiology laboratory. The MIC of all isolates was confirmed by the broth microdilution method. All isolates were identified to species level by MALDI-TOF MS Microflex (Bruker Daltonics). For all the experiments, the isolates were subcultured onto a Mueller-Hinton agar plate overnight at 35 ± 2°C to provide fresh cultures for the assays.

### Carbapenemase detection and standard susceptibility testing.

The presence of the carbapenemase genes was previously evaluated in all isolates by high-resolution melting real-time PCR (qPCR-HRM), using a multiplex assay with primers for *bla*_KPC_, *bla*_NDM_, *bla*_OXA-48-like_, *bla*_IMP_, *bla*_GES_, *bla*_VIM_, and *bla*_SPM-1_ genes, as previously described (31).

Broth microdilution, the standard method to establish meropenem MIC, was performed according to EUCAST (20, 32) using meropenem trihydrate (Eurofarma). The breakpoints (S, ≤2 mg/L; R, >8 mg/L) were used to interpret the results as “susceptible, standard dosing regimen” (S), “susceptible, increased exposure” (I), and “resistant” (R) (32). Quality control was performed with Escherichia coli ATCC 25922, according to EUCAST instructions (20).

### Antibiotic solution preparation.

For the adapted MBT-ASTRA, meropenem solution was prepared from commercially available meropenem disks (Oxoid Thermo Scientific). Each lot of meropenem disks was quality control checked, as recommended by EUCAST (20). In order to obtain 8 and 16 mg/L as final concentrations, one and two disks, respectively, containing 10 μg meropenem each, were eluted in 0.625 mL of distilled water for 10 to 15 min.

To evaluate the possibility that the antibiotic does not elute completely in 10 to 15 min or to account for a possible variation between preparations, we performed broth microdilution with 2 solutions prepared with disks eluted after 10 to 15 min and 2 solutions eluted after 2 h, in duplicates, with Escherichia coli ATCC 25922.

### Adapted method of MBT-ASTRA.

The method presented here was adapted from the work of Lange et al. (11). Isolates were suspended in brain heart infusion (BHI; Kasvi) broth to obtain a concentration of an 0.5 McFarland standard. A volume of 100 μL was added to two microtubes: one containing 100 μL of distilled water and one with 100 μL of meropenem solution. The microtubes were vortexed and incubated, without agitation, for 2 h at 35 ± 2°C. Then, the suspensions were centrifuged at 16,060 × *g* (13,000 rpm) for 2 min. The supernatant was discarded, and 150 μL of distilled water was added to the microtubes. The microtubes were vortexed again and centrifuged at 16,060 × *g* (13,000 rpm) for 2 min. The supernatant was discarded, and 100 μL of 70% ethanol (Merck) was added to each microtube. The microtubes were vortexed, left to rest at room temperature for 5 min, and centrifuged at 16,060 × *g* (13,000 rpm) for 2 min. The supernatant was discarded, and the pellet was left to air dry at room temperature. Subsequently, 10 μL of 70% formic acid (Sigma-Aldrich) and 10 μL of 100% acetonitrile (Merck) containing an internal standard (RNase B, 20 g/L; Sigma-Aldrich) were added, and the microtubes were vortexed and centrifuged at 16,060 × *g* (13,000 rpm) for 2 min. Then, 1 μL of the supernatant was spotted, in quadruplicates, onto a polished steel target plate and left to air dry. Afterward, 1 μL of α-cyano-4-hydroxycinnamic acid (HCCA; Bruker) at 10 mg/mL in 50% acetonitrile and 2.5% trifluoracetic acid (Sigma-Aldrich) was added, and the spots were left to air dry at room temperature.

For spectrum acquisition a Microflex LT (Bruker Daltonics) mass spectrometer with flexControl 3.4 software (Bruker Daltonics) was used. The parameters applied were the same ones used for microbial identification (ion source 1, 20 kV; ion source 2, 18.25 kV; lens, 6 kV; detector gain, 2,850 V), ranging from 2,000 to 20,000 Da (method named MBT_FC.par, as set by the manufacturer). The mass spectrometer was externally calibrated with bacterial test standard (BTS; Bruker), according to the manufacturer’s instructions. For automated acquisition of the spectra, MBT_AutoX automated programming was used.

**FIG 3:**
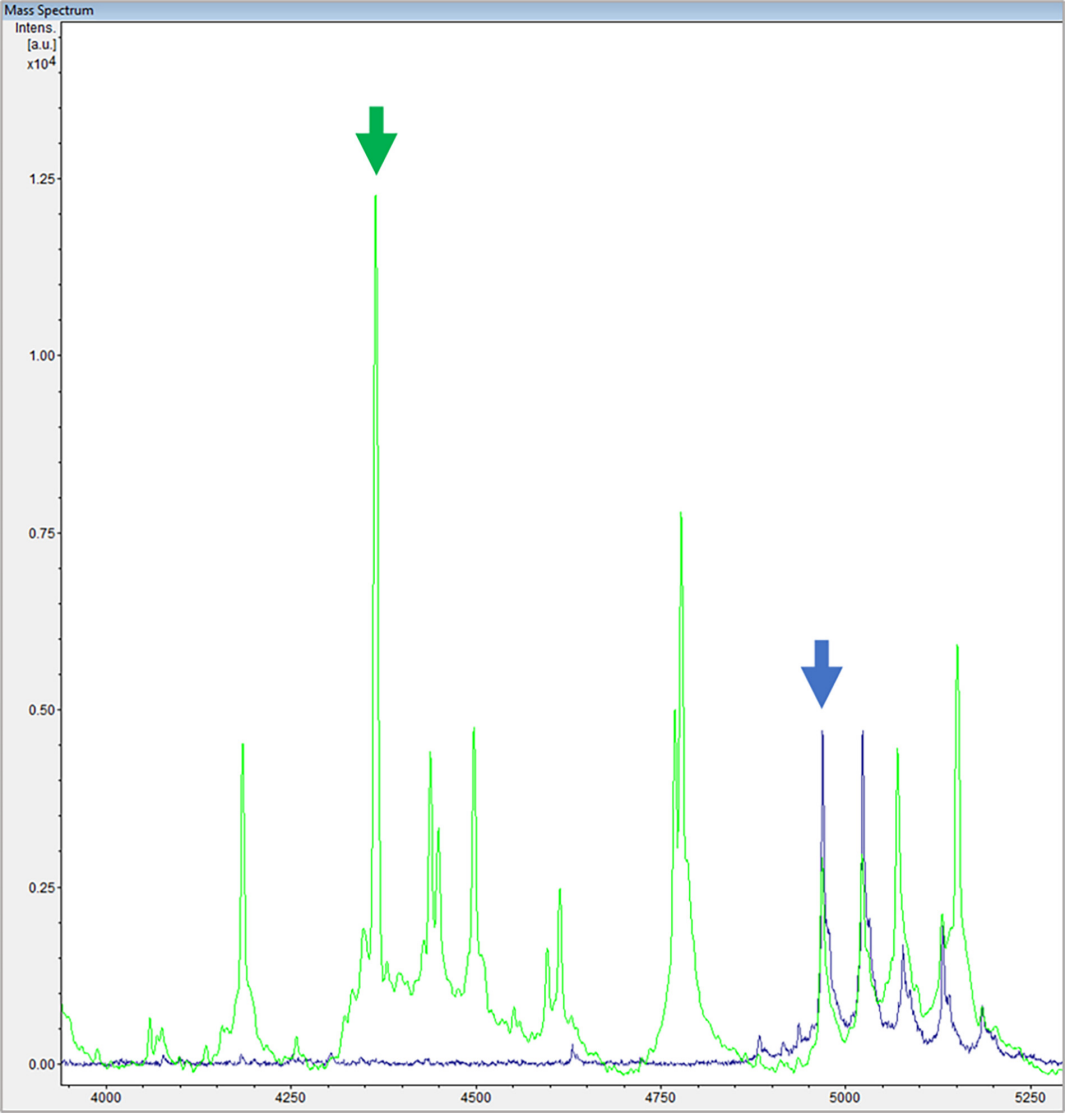
Spectrum from bacteria with internal standard (green) overlaid with the internal standard spectrum alone (blue). The green arrow indicates a peak exclusive of the bacterium as it is present only in the green spectrum. The blue arrow indicates an internal standard peak, because it is present in both spectra. Intens., intensity; a.u., arbitrary units.

Subsequently, spectra were analyzed with flexAnalysis 3.4 software (Bruker Daltonics). For each spectrum 6 peaks were manually selected (3 peaks from the bacterium and 3 peaks from the internal standard), and their intensities and AUC were annotated according to the raw spectrum and after spectrum processing (baseline subtraction and smoothing). Peaks from bacteria were selected in the following ranges: 4,000 to 5,000, 6,000 to 6,300, and 9,000 to 10,000 *m/z*. Peaks from the internal standard were approximately 4,970, 7,454, and 14,910 *m/z*. The peaks from the bacteria were carefully selected after comparison with the spectrum of RNase B alone in order to avoid mistakenly choosing peaks from the internal standard (Fig. 3). Spectral data were exported as .xlsx files. For each quadruplicate, the sum of the intensities or the sum of the AUCs from the bacterium (Int_Bac_ or AUC_Bac_) was normalized with the sum of the intensities or the AUCs from internal standard (Int_RNase B_ or AUC_RNase B_). Then, RG value was calculated by the ratio of the median of the normalized peaks from bacteria incubated with antibiotic (Int_ATB_ or AUC_ATB_) to the median of the normalized peaks from bacteria incubated without antibiotic (Int_BHI_ or AUC_BHI_). Normalization and RG value can be mathematically demonstrated as follows: RG = median (∑ Int_Bac+ATB_/∑ Int_RNase B+ATB_)/median (∑ Int_Bac+BHI_/∑ Int_RNase B+BHI_) or median (∑ AUC_Bac+ATB_/∑ AUC_RNase B+ATB_)/median (∑ AUC_Bac+BHI_/∑ AUC_RNase B+BHI_).

In order to establish a cutoff value to classify the isolates into S, I, and R, a receiver operating characteristic (ROC) curve was constructed for each combination of the parameters used (intensity and AUC) and the spectrum (raw and processed). First, the ROC curve was constructed to separate S from I and R isolates and then S and I from R isolates. ROC curves also provided sensitivity and specificity, which were used to evaluate whether peak intensity and AUC could be equally used to calculate RG values and whether there was any difference by applying or not applying spectrum processing. The area under the ROC curve was also provided.

Considering intensity and processed spectrum, categorical agreement, minor discrepancy, major discrepancy, and very major discrepancy were calculated according to FDA guidance (33). Additionally, Spearman’s test was applied to evaluate the correlation between RG value and meropenem MIC. We also used RG values recalculated with 4 peaks: 2 from bacteria in the ranges 6,000 to 6,300 and 9,000 to 10,000 *m/z* and 2 from the internal standard, approximately 7,454 and 14,910 *m/z*. Using these recalculated RG values with 4 peaks, new cutoff values were determined by ROC curve.

### Statistical analysis.

RG values were calculated using Excel software (Microsoft), and statistical analyses were performed with PASW Statistic 18.0.0. For ROC curve and Spearman’s test, 95% and 99% confidence levels were used, respectively.
